# Supplementary material for: Coexisting maternal and child undernutrition in Ethiopia: Spatial modeling and multilevel analysis of consecutive EDHS data
Source: PLoS One. 2026 Jan 27;21(1):e0341870. doi: 10.1371/journal.pone.0341870 (PMC12843538; doi:10.1371/journal.pone.0341870)
Supplement: Attachment — Submitted filename: review report.docx [file pone.0341870.s001.docx]

**Review Report of the manuscript titled “Coexisting Maternal and Child Undernutrition in Ethiopia: Spatial Modeling and Multilevel Analysis of Consecutive EDHS Data.”**

The reviewer appreciates the authors for their efforts in tackling the important and very pertinent public health problem of concurrent undernutrition in Ethiopian mothers and children. Using geographic modeling, multilevel analysis, and many rounds of EDHS data (2000–2016) offers important insights into long-term trends and regional differences. It is praiseworthy that geospatial methods and hierarchical models have been combined, and this has great potential to guide focused actions and policy development. Overall, the paper addresses a significant issue with a big dataset; nonetheless, a number of significant changes are required to improve methodological rigor and clarity. In order to improve the manuscript's representation in front of a global audience and ensure that it complies with PLOS ONE standards, the authors are asked to make the following major as well as minor changes.

1. The abstract (Lines 27–60) is unnecessarily long and includes repetitive background information. It would be much stronger if it were shortened to the essential elements: the main objective, a brief method summary, the key findings, and a final implication sentence.
2. The introduction (Lines 72–122) contains several overlapping explanations about global undernutrition. These paragraphs should be merged and streamlined so that the central research gap in the Ethiopian context becomes clearer.
3. The spatial analysis component (Lines 185–209) needs more methodological depth. The manuscript should include the formulas and proper citations for Moran’s I and Getis–Ord Gi*, and it should also clarify the type of spatial weights matrix used, the variogram model selected for kriging, and the statistical settings applied in SaTScan.
4. The multilevel modelling section (Lines 210–244) would benefit from additional clarity. Introducing the actual model equation, noting whether DHS sampling weights were incorporated, and briefly explaining how multicollinearity and variable selection were handled would strengthen this part.
5. Figure 3 (Lines ~289–294) is of low resolution and appears pixelated. It should be replaced with a minimum 300-dpi version that has clearer boundaries, a visible scale bar, a north arrow, and a more readable legend.
6. Figure 4 (Lines 295–303) also lacks visual sharpness and color clarity. A higher-resolution version with cleaner symbology and clearer regional labels is needed for publication quality.
7. Figure 5 (Lines 305–312), which presents the hotspot analysis, is blurry. It should be regenerated with higher resolution and should include proper cartographic elements like a scale bar, north arrow, and a more legible legend.
8. Figure 6 (Lines 313–320), the kriging interpolation map, appears pixelated and the color gradient is uneven. A smoother, higher-quality rendering with improved symbology would greatly enhance interpretability.
9. Figure 7 (Lines 321–330), showing the SaTScan clusters, is difficult to read because of overlapping cluster circles. This figure should be redrawn with clearer marker sizes, improved separation, and a refined legend.
10. The reported sample size is inconsistent between the Methods (Lines 38–44) and the Results (Lines 44–50). The manuscript should present one consistent figure throughout.
11. The results section (Lines 270–330) is too descriptive in several places. Presenting the findings in a more succinct, quantitative manner would improve readability.
12. The discussion (Lines 364–425) frequently restates the results instead of interpreting them. This section would be stronger if it focused more on explaining the findings, connecting them to previous research, and examining underlying mechanisms.
13. The conclusion (Lines 426–433) could be enhanced by adding a few explicit directions for future work, such as incorporating environmental variables, dietary diversity indicators, or more advanced spatial modelling approaches like GWR or MGWR.
14. Several typographical and referencing issues appear throughout the manuscript (e.g., Lines 92, 185, 572). These include spelling errors, inconsistent EDHS notation, and the use of non-academic sources like Wikipedia, which should be replaced with authoritative references.
15. The operational definition of CMCU (Lines 165–169) needs stronger justification. In particular, using maternal height under 155 cm as a measure of undernutrition should be supported with literature, and a sensitivity analysis would be advisable since height reflects long-term rather than current nutritional status.
16. Although the dataset spans from 2000 to 2016, temporal patterns are not adequately explored. Including a brief trend analysis or highlighting shifts across survey years would strengthen the interpretation.
17. The manuscript should acknowledge the effect of DHS geographic displacement on spatial accuracy, especially regarding Moran’s I, Gi*, SaTScan, and kriging. This is an important methodological limitation that should be mentioned.
18. The discussion would benefit from incorporating environmental and agro-ecological context. Interpretation of spatial clusters should consider factors such as drought frequency, agricultural productivity, and dominant livelihood systems in each region.
